# A Quality Improvement Study to Improve Oxygen Prescription in the PICU of a Tertiary Care Centre in Delhi, India

**DOI:** 10.7759/cureus.43332

**Published:** 2023-08-11

**Authors:** Anushruti Shukla, Vikram Bhaskar, Prerna Batra

**Affiliations:** 1 Medical School, University College of Medical Sciences, Delhi, IND; 2 Pediatrics, University College of Medical Sciences, Delhi, IND; 3 Pediatrics, University College of Medical sciences, Delhi, IND

**Keywords:** oxygen delivery in neonates, attitude of health personnel, quality assessment in healthcare, prescription audit, pdsa cycle, oxygen therapy, paediatric intensive care unit, quality improvement projects

## Abstract

Introduction

Oxygen has been gravely misused since its inception as a therapeutic agent. There is a deficit of audits and baseline data for the Indian population, especially in the pediatric age group, which doesn’t allow for standardization of protocols and guidelines.

Objective

Our study aimed at increasing valid prescription rates to 90% by implementation of quality improvement interventions, and assessing knowledge and perception of healthcare workers towards oxygen therapy.

Methodology

It followed a before-and-after prospective observational study model where baseline audit data was compared with data observed after the implementation of quality improvement strategies. The data was collected through an audit of the medical records of all pediatric patients receiving oxygen therapy in the PICU. Knowledge and perception of healthcare workers about oxygen therapy were assessed via a self-designed questionnaire. The study was undertaken in three phases, including Quality Improvement (QI) team formation and data collection, root cause analysis, and implementation of Plan-Do-Study-Act (PDSA) cycles.

Observations and results

In the baseline audit, 1.4% of the prescriptions were complete and valid. Subsequently, over the course of four PDSA cycles, valid prescription rates increased; 62.07% in the first, 79.51% in the second, 81.81% in the third, and 91.42% in the fourth cycle respectively. After applying the chi-square test to compare PDSA4 and baseline data, the p-values for written prescriptions and target saturation were found to be statistically significant.

In the healthcare worker survey, we found that 100% of them were aware of indications of oxygen prescription, FiO2, and side effects of excessive usage of oxygen therapy, 95% were aware of conditions affecting pulse-oximetry in the pediatric age group, and 75% knew about target saturation and its significance and the procedure to change alarm settings on the monitor.

Conclusion

Currently, there exists a lack of effective oxygen prescription audits, especially in India, which can be attributed to a lack of awareness and partly, a lack of initiative. Quality improvement initiatives are effective in improving the valid oxygen prescription rate. However, sustained goals can be achieved through regular audits only.

## Introduction

Oxygen is a commonly used drug in the clinical setting; however, it still remains poorly prescribed and injudiciously used, even after more than 200 years of its inception as a therapeutic agent [1]. The indication for oxygen therapy is mainly hypoxemia, and not breathlessness [2]. Hypoxemia is suspected in cases of central cyanosis, respiratory distress, and low saturation levels assessed via pulse oximeter. The adverse effects of this condition are well-documented [3]. Another extreme, oxygen toxicity, or hyperoxemia, has also been found to be associated with a higher risk of mortality, owing to free-radical injury [3-5]. In patients with retention of carbon dioxide, as in COPD (chronic obstructive pulmonary disease), over-oxygenation can precipitate the development of iatrogenic hypercapnia [6]. In the case of pre-term neonates, broncho-pulmonary dysplasia and retinopathy of prematurity are seen as a consequence of hyperoxia [7]. Another consequence of unregulated oxygen administration is wastage and extra expenses. Thus, there is a need to regulate oxygen prescription and administration [8].

The British Thoracic Society (BTS) is the pioneer in the standardization of protocol for oxygen prescription. The first set of guidelines came in 2008 [9], and has been followed by yearly audits to analyze improvements. The majority of studies done internationally highlight the sub-optimal adherence to these guidelines [10,11]. The 2015 BTS audit has shown that 42.5% of patients who were on oxygen did not have a valid prescription [12]. For the purpose of this study, we define valid prescriptions in terms of a modification of the BTS guideline. It comprises four documented items in written order: mode of delivery, flow rate, target saturation (BTS-recommended), and an additional parameter of FiO2 [10].

Unfortunately, in the Indian scenario, there is a serious deficit of knowledge in this regard, due to the lack of audits and thus almost non-existent baseline data. As a result, prescription protocols are limited. Furthermore, there exist no guidelines for the pediatric population.

The updated BTS guidelines [13], which are the most widely accepted, are for ages 16 and above.

A number of quality improvement projects have been undertaken in clinical settings and are found to be efficient in increasing compliance with oxygen prescription guidelines [1]. They are yet to be done for the Indian population.

## Materials and methods

Design

After analyzing the problem at hand, we decided to constitute a Quality Improvement (QI) Team consisting of one resident doctor, one staff nurse, and one nursing orderly. Due to the lack of any previous oxygen prescription audits, we planned to conduct an audit of prescriptions for establishing baseline data before moving forth. A self-designed questionnaire was given to healthcare workers to assess their knowledge of oxygen and its prescription practices. After the analysis of baseline data, the root cause analysis was performed via the method of fishbone analysis. According to the deficits identified, the QI team devised a plan for interventions. After implementing them, data was collected again for auditing. On the basis of the results, the QI team either accepted, rejected, or modified the intervention. The same procedure was followed after their implementation. The entire project was undertaken after clearance from the institutional ethics committee (IEC-HR).

Patient and public involvement

Our study included reviewing medical records and patients were not directly involved. The study was approved by the IRB of our institution.

Measurement

We calculated our sample size after considering an alpha error of 5%, and power of 90%, which came out to be 36 in both the before and after groups. However, we were able to audit a much larger sample size of 70 in our baseline data. Baseline data was collected over a period of seven days in the PICU. The medical records of all the patients admitted to the PICU who were receiving oxygen were audited, and the variables that were recorded included the following: a written record of oxygen prescription, mention of oxygen delivery system, mention of desired flow rate, mention of the fraction of inspired oxygen concentration (FiO2), and mention of target oxygen saturation. 

The baseline data also included the evaluation of knowledge of nurses and doctors regarding oxygen prescription, using a self-designed questionnaire. A total of 20 responses were collected (12 nurses, four senior residents, four junior residents).

All the data was entered into an Excel sheet and was analyzed to calculate baseline data for adequacy of oxygen prescription.

Strategy

Our SMART aim (specific, measurable, achievable, relevant, and time-bound aim) was to increase the rate of valid oxygen prescriptions to 90% in the PICU. We aspired to achieve it by implementing and analyzing Plan-Do-Study-Act (PDSA) cycles. A total of four PDSA cycles were implemented.

PDSA Cycle 1

We counseled the healthcare workers of the PICU in a 20-minute session. They were made aware of the indications of oxygen prescription and its ill effects in cases of over-prescribing.

PDSA Cycle 2

We introduced the second intervention in the form of a poster on oxygen prescription SOPs (standard operating procedures) pasted on the PICU walls. The posters were displayed in areas with maximum visibility and acted as a constant reminder to those prescribing oxygen.

PDSA Cycle 3

A stamp was introduced to be put on all prescriptions with the following details: date, device, flow rate (in the case of non-ventilated patients), FiO2 (in the case of ventilated patients), and target saturation.

PDSA Cycle 4

A decision was made to modify the intervention with a stamp to a monitoring sheet to be placed at the bedside of patients. The sheet had the following parameters mentioned, date, device, flow rate (in case of non-ventilated patients), FiO2 (in the case of ventilated patients), and target saturation. Prescribers were instructed to fill in the sheet whenever they changed any of the parameters in the prescription.

## Results

Baseline data

Prescription Audit

For the baseline data collection, a total of 70 prescriptions of patients in the PICU were audited and evaluated for the following parameters: written prescription for oxygen, oxygen delivery system, flow rate, FiO2, and target saturation.

We found that out of the total number of prescriptions, only 50% (n=35) had a written record of oxygen therapy. In the remaining 50%, oxygen therapy was mentioned in the patient’s file either in the monitoring chart or elsewhere or it was not mentioned at all. The oxygen delivery system was written in 100% of the prescriptions. The flow rate was mentioned in 62 prescriptions (88.6%) while FiO2 was mentioned in 54 (77.1%) prescriptions. Target saturation was mentioned in only one prescription (1.4%).

Healthcare Professional Survey

We surveyed healthcare professionals using a self-designed questionnaire, to assess their knowledge about oxygen prescription practices. All the survey-takers were aware of indications of oxygen prescription, FiO2, and side effects of excessive usage of oxygen therapy. Out of these 20 healthcare workers (12 nurses and eight resident doctors), 95% were aware of conditions affecting pulse-oximetry in the pediatric age group. Although these healthcare workers kept on rotating in different shifts, the pool of healthcare workers remained the same for the duration of the study. Only 75% of the healthcare workers knew about target saturation and its significance, and the procedure to change alarm settings on the monitor. The responses have been tabulated in Table 1.

**Table 1:** Healthcare Workers Survey Results

SNo.	Question	Response	
		Yes	No
1	Do you know the indications for prescribing oxygen to a patient?	20	0
2	Do you know the FiO2 delivered by various oxygen devices?	20	0
3	Are you aware of any side effects of excessive oxygen?	20	0
4	Are you aware of target oxygen saturation levels?	15	5
5	Do you know the procedure to change alarm settings in monitor?	15	5
6	Are you aware of any conditions that affect pulse-oximetry?	19	1

Root Cause Analysis

**Figure 1:**
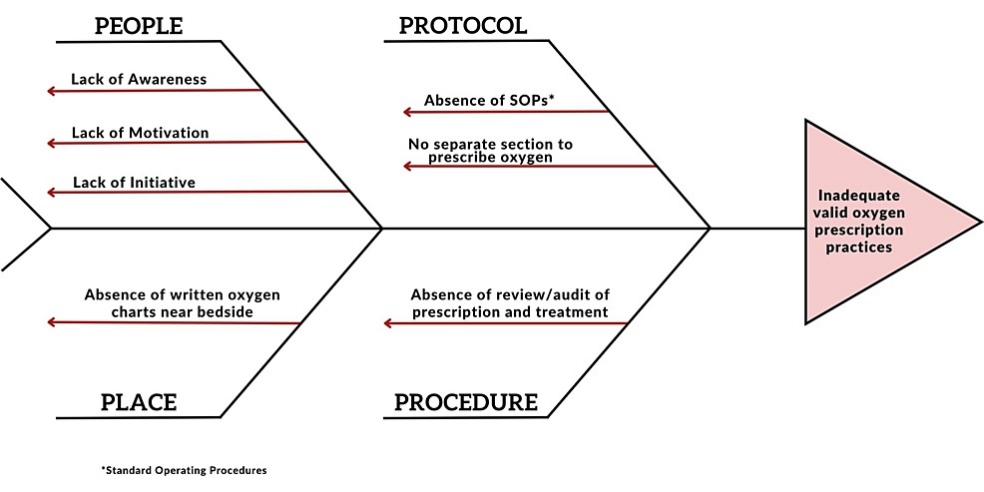
Root Cause Analysis (Fish-Bone Method) *standard operating procedures

The fishbone analysis revealed that there was a lack of awareness and motivation among both resident doctors and other healthcare workers regarding oxygen prescriptions. There was no written policy or SOPs, and there was no designated place in the file to mention oxygen prescriptions (Figure 1).

Quality Improvement Interventions

Four PDSA cycles were undertaken throughout the length of the project. From the baseline (1.4%) valid prescription rates increased to 62.07%, 79.51%, 81.81%, and 91.42% in cycles 1, 2, 3, and 4 respectively.

PDSA Cycle 1 

Twenty-nine files were analyzed. An increase in percentage was noted in all the parameters. A valid, written prescription was found in 62.07% of files. O2 delivery device, flow rates, and FiO2 were mentioned in 100% of the files. The target saturation was mentioned in 65.51% of prescriptions. Table 2 shows the obtained data.

**Table 2:** PDSA cycle 1 (counseling session)

Date	Number of Files	Written Prescription (%)	Device (%)	Flow Rate (%)	FiO2 (%)	Target Saturation (%)
23/09/2022	7	57.14	100	100	100	71.42
24/09/2022	8	75	100	100	100	75
25/09/2022	7	71.42	100	100	100	71.42
26/09/2022	7	42.85	100	100	100	42.85

PDSA Cycle 2 

Out of a total of 83 files, 83.13% had a complete written prescription. The device of oxygen delivery was mentioned in 100% of the files. The flow rate was mentioned in 95.83% of the 24 files of non-ventilated patients and the FiO2 was mentioned in 96.61% of the 59 files of ventilated patients. Around 79.51% of prescriptions had a mention of target saturation. Table 3 summarises the findings.

**Table 3:** PDSA cycle 2 (SOPs as posters on the walls of PICU)

Date	Number of files	Written Prescription (%)	Device (%)	Flow Rate (%)	FiO2 (%)	Target Saturation (%)
27/09/2022	6	100	100	100	100	83.33
28/09/2022	12	66.66	100	75	100	75
29/09/2022	5	80	100	100	100	80
30/09/2022	4	75	100	100	100	75
1/10/2022	5	75	100	100	100	75
2/10/2022	5	60	100	100	100	66.66
3/10/2022	5	80	100	100	100	80
4/10/2022	5	100	100	100	100	80
5/10/2022	5	100	100	100	100	100
6/10/2022	4	75	100	100	100	75
7/10/2022	9	88.88	100	100	100	66.67
8/10/2022	6	83.33	100	100	100	83.33
9/10/2022	12	91.66	100	100	75	100

PDSA Cycle 3 

A total of 33 files were audited, out of which 84.84% had a written prescription, with the rest having it mentioned in other parts of the file. All (100%) files had records of the device used, and all seven non-ventilated patients had their flow rates mentioned in the files. Around 96.15% of the patients out of a total of 26 ventilated patients had FiO2 mentioned in their files. Target saturation was present in 81.81% of these files. This data is summarised in Table 4.

**Table 4:** PDSA cycle 3 (stamp on prescription sheets)

Date	Number of Files	Written Prescription (%)	Device (%)	Flow Rate (%)	FiO2 (%)	Target Saturation (%)
10/10/2022	6	66.66	100	100	100	66.66
11/10/2022	7	85.71	100	100	100	85.71
12/10/2022	6	83.33	100	100	100	83.33
13/10/2022	6	83.33	100	100	100	83.33
14/10/2022	8	75	100	100	100	75

PDSA Cycle 4 

Of a total of 35 files that were taken for analysis, 94.28% of files contained a written oxygen prescription, whereas the remaining files have evidence of oxygen prescription elsewhere. The device was mentioned in 100% of the files. Flow rate and FiO2 were also mentioned in 100% non-ventilated (5) and ventilated (30) patient files respectively. Target saturation increased to 91.42%. The data presented is tabulated in Table 5.

**Table 5:** PDSA cycle 4 (record of oxygen prescription on separate sheet)

Date	Number of Files	Written Prescription (%)	Device (%)	Flow Rate (%)	FiO2 (%)	Target Saturation (%)
15/10/2022	6	100	100	100	100	100
16/10/2022	5	100	100	100	100	100
17/10/2022	5	100	100	100	100	80
18/10/2022	5	100	100	100	100	100
19/10/2022	4	100	100	100	100	100
20/10/2022	5	80	100	100	100	80
21/10/2022	5	80	100	100	100	80

**Figure 2:**
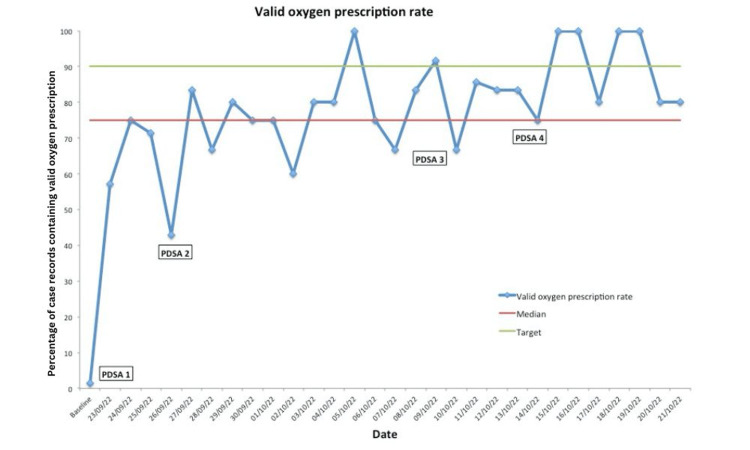
Run Chart - Valid Oxygen Prescription Rate

The run chart plot for the valid oxygen prescription rate is shown in Figure 2. It shows six points plotted above the target line, indicating the sustainability of our results. 

Statistical analysis

SPSS software v. 23 (IBM Corp., Armonk, NY) was used for data management and analysis. The chi-square test comparing baseline and post-PDSA4 data was analyzed. The p-values for written prescription, FiO2, device, target saturation, and flow rate were 0.007, 0.084, 0.99, <0.001, and 0.128 respectively. Values lesser than 0.05 were taken to be statistically significant (Table 6).

**Table 6:** Comparison of mean percentages of various components of oxygen prescription following each PDSA cycle *Chi-square test comparing baseline and post-PDSA4 data

	Baseline	PDSA1	PDSA2	PDSA3	PDSA4	P Value*
Written Prescription	50	62.06	80.7	78.7	93.3	0.007
FiO2	77.1	100	96.3	100	100	0.084
Device	100	100	100	100	100	0.99
Target Saturation	1.4	65.5	81.9	78.7	90	<0.001
Flow Rate	88.6	100	96.3	100	100	0.128

## Discussion

With this project, we aimed to increase valid oxygen prescription rates in our institution, from the baseline to 90%. This was a realistic and achievable aim and was agreed upon after extensively researching similar projects undertaken in the past in comparable settings. Our priority remained coming up with solutions that were effective and sustainable, instead of serving short-term.

During the formation of the QI team, we were aware of the contributions of additional personnel other than the prescribing doctor. Therefore, an active attempt was made to include the maximum number of healthcare workers, including consultants, junior residents, senior residents, and the nursing staff. Everyone had their own viewpoints to offer owing to their varied years of experience, personal opinions, and the nature of their jobs. This helped us in understanding the existing shortcomings multi-dimensionally.

For our first intervention, the QI team decided to counsel the healthcare personnel working in the PICU. In a 20-minute session, they were made aware of the indications of oxygen prescription, its ill effects in cases of over-prescribing, and the quality improvement project and its objectives. It was observed that just after counseling, there was a huge increment in each parameter of the oxygen prescription and an overall increase in the valid prescription rate; however after a short period of four days, the effect started to wear off, and the rates started falling. This can be attributed to the transient nature of the effect of counseling, as is seen in most quality improvement exercises [14]. This intervention was thus rejected due to issues with sustenance.

**Figure 3:**
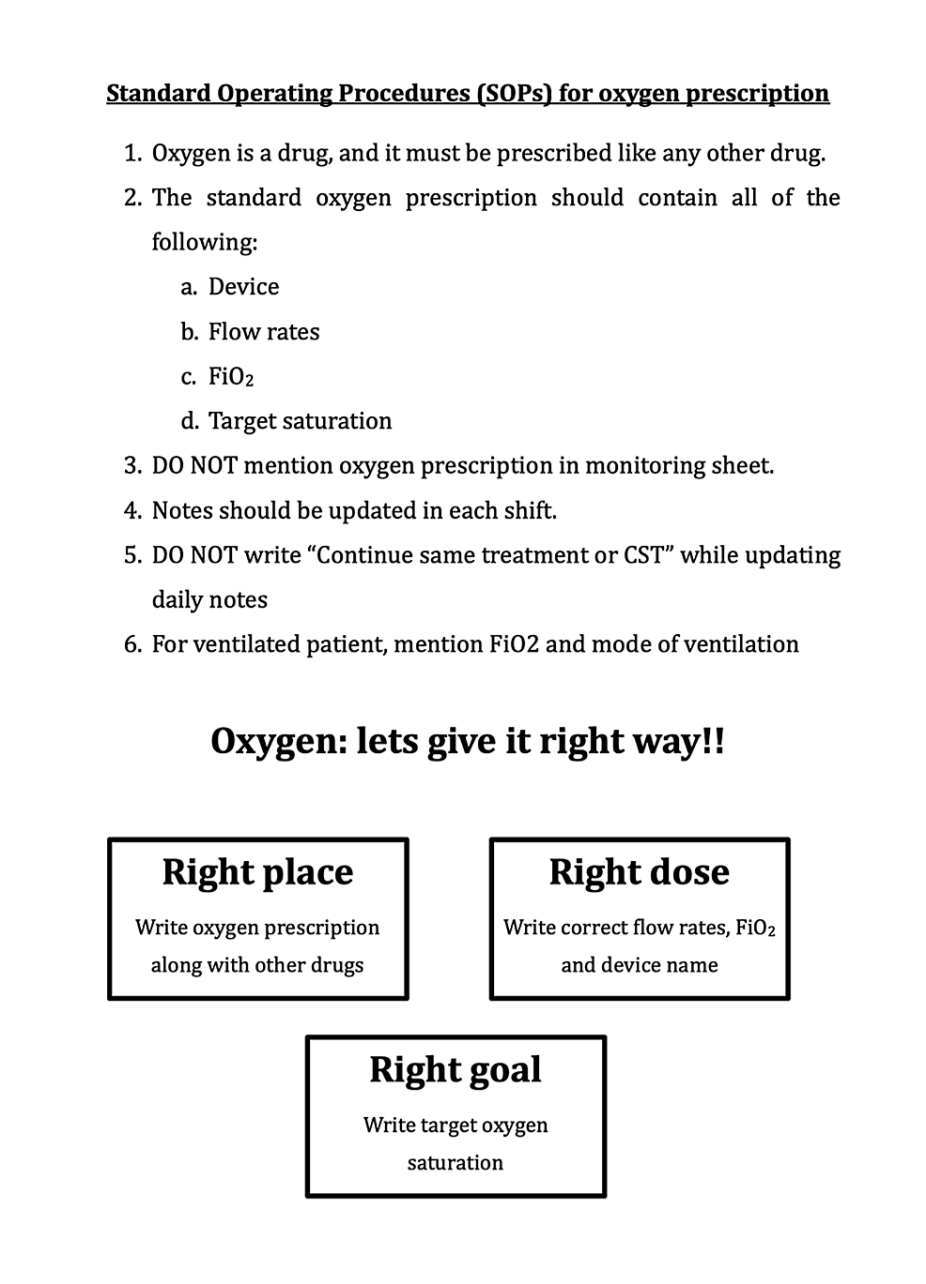
Standard Operating Procedures (SOPs) for Oxygen Prescription

The second intervention decided was the drafting of the SOPs for oxygen prescriptions in the PICU. Two copies of this document were pasted as attractive posters on the PICU walls, after understanding which areas were more observable than others (Figure 3). An increase in valid prescription rates was observed. Target saturation, which happened to be one of the least mentioned parameters on prescriptions during the baseline analysis, was mentioned in 100% of prescriptions on a few days in this period. It was noted that on some days, the written prescription was not present or target saturation was missing, which could be attributed to a particular set of personnel, who had not been a part of the counseling, and thus often missed the posters on the walls. This dip is very apparent on two occasions during the audit period. However, the intervention as a whole was judged successful and was accepted. The SOPs have proven to be sustainable and have results independent of the staff at work [15, 16].

**Figure 4:**
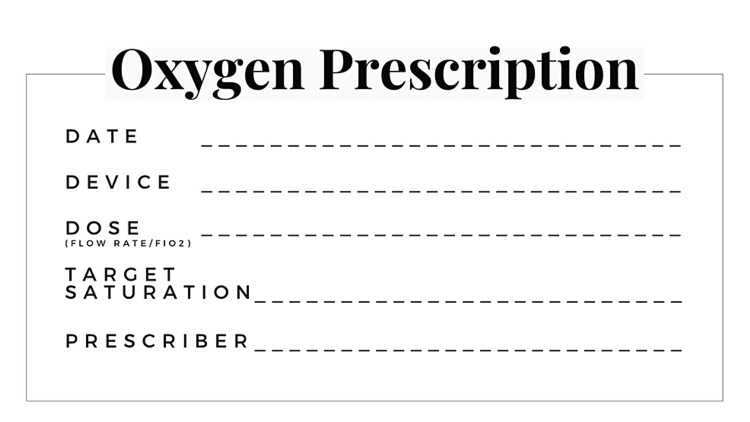
Oxygen Prescription Stamp

A third intervention executed was having oxygen prescription stamps on the prescriber’s sheets. The audit period showed an increase in various parameters, but there was an issue of feasibility and sustenance in the long term. The QI team, after consulting the attending physicians, made a decision to modify this intervention and replace it with a more easy-to-use bedside record sheet (Figure 4). 

The final PDSA cycle was implemented with the bedside oxygen prescription record sheet, with all relevant parameters. Prescribers were instructed to fill in the sheet whenever they changed any of the parameters in the prescription. This intervention showed the highest sustenance. A maximum increase in all parameters was noted. The record sheet served as a recurrent reminder for prescribers to mention all parameters on their prescriptions, which was an effective strategy.

In a 16-week quality improvement project with eight PDSA cycles conducted in a general hospital in Qatar, documentation of indication of oxygen therapy and target saturation improved to 85% and 86%, from a baseline of 75% and 47% respectively. Interestingly, there was a significant decline in the documentation of target saturation levels when a new team of residents joined [17]. A similar finding was observed in our study, particularly in the period of PDSA cycle 2. 

Promising results are also seen in rural settings, for instance, in a quality improvement project in New South Wales where initially, only 2.4% of patients had a complete oxygen prescription specifying target saturation, device, and flow rate. The interventions lead to an increase of 34% in prescriptions (χ2 = 56.88, df = 5, P < 0.0001) [18].

In a general hospital quality improvement project, there was a rise in valid prescribing rates to 66% from the baseline (14%) even though the target was 95%. In order to counteract this shortcoming, regular updates on e-modules became mandatory to keep spreading authentic information in relation to oxygen prescriptions [19].

Other interventions have also been reviewed by researchers in their hospitals. In a study from Bristol, the planned intervention of nurse-facilitated reminders led to a significant increase in valid prescription rates from 0% pre-intervention to 49% post-intervention (p < 0.0001) [20]. In another study from the UK, their planned interventions included a new chart in the patient file specifically for oxygen, oxygen ‘alert’ stickers, and MEWS (modified early warning system) charts. Both valid prescription rates and target saturation mentions grew to 94% from the initial 55% and 50% respectively [16]. 

The awareness and initiative of healthcare workers is of utmost importance. In our survey, the majority knew about the basic indications of prescription, FiO2, and pulse oximetry, but had difficulty understanding target saturation and the settings on alarm monitors. In this regard, one group of researchers did a FRAM (Functional Resonance Analytic Method) analysis to understand the variability of prescription practices attributed to attitudes, behavior, and knowledge of doctors. They found that many prescriptions are often not doctor-led. The nurses often demanded guidance corresponding to said prescriptions [21].

Through this quality improvement project, we achieved a tremendous increase in valid and complete prescription rates, from 1.4% to 91.42%. However, it had its set of limitations. There is still scope for further improvement in prescription practices. It would be prudent to come up with small but sustainable changes in infrastructure and protocols to promote valid prescription practices. All stakeholders including healthcare personnel, patients, and administrative workers share the duty of discussing possible solutions to promote positive change.

Limitations

A recurrent challenge we faced was the rotation of residents in the PICU on a regular basis. Instructions had to be repeated to the teams, and if some personnel missed out, their performance negatively impacted the overall record due to non-compliance. We also felt that owing to the short study period, we couldn’t effectively gauge the sustainability of our interventions. Periodic prescription audits might be the way forward in this regard. We recognize that the interventions we illustrated might not work out in other settings due to differences in infrastructure, administrative practices, and resources available. Even in our current setting, it would be ill-informed to suggest that these were permanent solutions, given the dynamic nature of healthcare settings. 

## Conclusions

The QI team was successful in increasing the valid oxygen prescription rates in our hospital from the baseline to the initially targeted 90%, with the biggest increment in the records of ‘target saturation’. The healthcare workers were also successfully sensitized about safe oxygen prescription practices, which translated into better compliance with established prescription protocols. We found that continuous reinforcement and periodic reminders contributed the most to improved outcomes. We also acknowledged the challenge of maintaining and further enhancing our progress. Therefore, we have aimed at instituting cost-effective yet sustainable changes in the PICU. We also identified that the lack of regular audits is the main hurdle. 

Given that healthcare institutions are dynamic settings, our future goals would be to modify our current interventions and develop new strategies when required. We wish to encourage other institutions to come up with tailored interventions in accordance with the challenges they face. 

## Appendices

Healthcare workers' survey questionnaire

Below is a template of the healthcare workers' survey questionnaire (Table 7):

AGE: SEX: OCCUPATION: YEARS OF EXPERIENCE:

**Table 7:** Healthcare Workers Survey Questionnaire

SNo.	Question	Response	
		Yes	No
1	Do you know the indications for prescribing oxygen to a patient?		
2	Do you know the FiO2 delivered by various oxygen devices?		
3	Are you aware of any side effects of excessive oxygen?		
4	Are you aware of target oxygen saturation levels?		
5	Do you know the procedure to change alarm settings in monitor?		
6	Are you aware of any conditions that affect pulse-oximetry?		
